# Mechanical tricuspid valve thrombosis in pregnancy: a case report and literature review on anticoagulation challenges and fetal protection strategies

**DOI:** 10.3389/fped.2025.1596199

**Published:** 2025-07-02

**Authors:** San Zhu, Can Luo, Bing Luo, Yaoyao Zhang, Qiang Wei

**Affiliations:** ^1^ Department of Obstetrics and Gynecology, West China Second University Hospital, Sichuan University, Chengdu, China; ^2^ Key Laboratory of Birth Defects and Related Diseases of Women and Children, Sichuan University, Ministry of Education, Chengdu, China

**Keywords:** mechanical valve thrombosis, maternal cardiac disease, anticoagulation therapy in pregnancy, fetal hemodynamic adaptation, multidisciplinary pregnancy care, prenatal environmental factors, long-term child health outcomes

## Abstract

**Background:**

Pregnancy in women with mechanical heart valves (MHVs) poses significant challenges in balancing maternal thromboprophylaxis and fetal safety. Anticoagulation strategies must simultaneously prevent life-threatening valve thrombosis and minimize fetal risks, yet optimal management remains controversial. While warfarin offers effective thromboprophylaxis, its embryotoxicity at higher doses (>5 mg/day) contrasts with low molecular weight heparin (LMWH), which lacks consensus on thrombotic efficacy despite fetal safety advantages.

**Case presentation:**

We report a case of a 30-year-old woman with mechanical mitral and tricuspid valves. She was maintained on low-dose warfarin (target INR 2.5–3.0) during early pregnancy. At 26–28 weeks of gestation, she developed exertional dyspnea; initial imaging showed stable valve function. At 33 + 2 weeks, worsening symptoms and echocardiographic evidence of tricuspid valve dysfunction prompted anticoagulation transition from warfarin to LMWH combined with vitamin K. After achieving an INR <1.4, cesarean delivery was performed at 33 + 3 weeks under general anesthesia, resulting in a live male infant without cardiac anomalies. Three days postpartum, mechanical tricuspid valve thrombosis with severe regurgitation was confirmed, necessitating bioprosthetic valve replacement on postoperative day 4. Maternal and neonatal outcomes were favorable.

**Conclusions:**

This case highlights the importance of individualized anticoagulation management, multidisciplinary coordination, and vigilant monitoring in optimizing outcomes for pregnant patients with MHVs. Tailored pharmacologic strategies represent key modifiable prenatal factors influencing both maternal safety and child health.

## 1 Introduction

Management of pregnancy in women with mechanical heart valves (MHVs) presents a critical challenge in balancing maternal thromboprophylaxis and fetal safety, particularly due to the heightened hypercoagulable state of pregnancy and teratogenic risks associated with anticoagulants (1, 2). Cardiovascular diseases complicate 1%–3% of pregnancies and account for 10%–15% of maternal mortality, with valvular pathologies significantly exacerbating risks through limited cardiac reserve (3, 4). For women with MHVs, anticoagulation strategies must navigate the dual imperatives of preventing life-threatening valve thrombosis and minimizing fetal harm, yet consensus on optimal regimens remains elusive (5, 6).

Warfarin remains the main anticoagulant for MHVs in non-pregnant patientsbut demonstrates dose-dependent embryotoxic, including congenital anomalies and fetal intracranial hemorrhage, particularly at doses exceeding 5 mg/day (7, 8). Low molecular weight heparin (LMWH) is often substituted in pregnancy to mitigate teratogenic risks; however, its efficacy in preventing valve thrombosis remains debated, with reports of thrombotic complications even under therapeutic anti-Xa monitoring (2, 9). The 2019 JACC review underscores the lack of high-quality evidence guiding anticoagulant selection, emphasizing the need for individualized risk-benefit assessments and multidisciplinary management (1).

The interplay between cardiac output redistribution and placental perfusion further complicates management. Right heart failure secondary to valve dysfunction reduces uterine artery flow, increasing risks of fetal growth restriction (10, 11). While LMWH may improve placental microcirculation in some high-risk pregnancies, recent randomized trials demonstrate no significant prolongation of pregnancy in cases of fetal growth restriction. It highlights the complexity of pharmacological interventions, including challenges related to dosing strategies, timing of administration, and inconsistent efficacy across different patient populations (9).

This case report examines a 30-year-old woman with MHVs who developed tricuspid valve thrombosis during pregnancy, necessitating anticoagulation transition from warfarin to LMWH and preterm delivery. By contextualizing this case within contemporary debates on LMWH safety, teratogen avoidance, and multidisciplinary protocols, we aim to contribute to the evolving discourse on perinatal pharmacotherapy optimization.

## 2 Case presentation

Written informed consent was obtained from the patient for the publication of this case. The patient, a 30-year-old woman with a history of two pregnancies (gravida 2, para 0) and one prior pregnancy loss, reported regular menstrual cycles, with her last menstrual period occurring on December 19, 2023. Eight years ago, at 16 weeks of gestation, she developed symptoms of fatigue and exertional dyspnea. Ultrasound revealed severe mitral stenosis and other cardiac abnormalities, necessitating mifepristone-induced abortion. Post-abortion, she was diagnosed with rheumatic heart disease (mitral stenosis with moderate tricuspid regurgitation) and underwent mechanical mitral and tricuspid valve replacement at a tertiary care center. Her recovery was uneventful, with no significant activity limitations.

**Figure 1:**
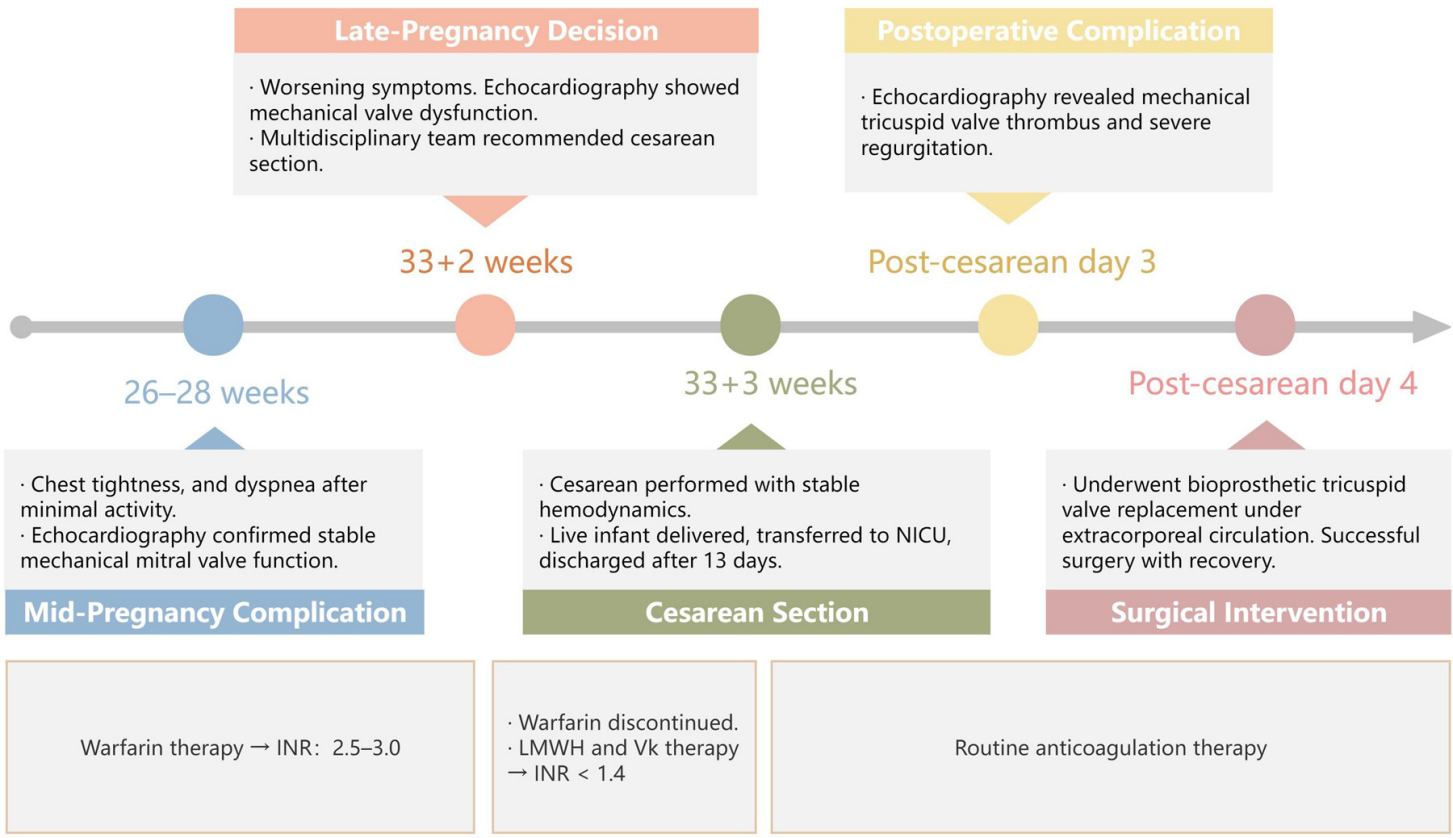
Timeline of the patient's current pregnancy process. LMWH, low-molecular-weight heparin; INR, international normalized ratio; Vk, vitamin K.

During her current pregnancy (Figure 1), she received regular cardiovascular follow-up, and maintained warfarin therapy (<2.5 mg/d) targeting an international normalized ratio (INR) of 2.5–3.0. At 26–28 weeks of gestation, she experienced chest tightness, and dyspnea after walking less than 100 meters, with symptoms resolving after 30 min of rest. She was admitted to the emergency department, where cardiac ultrasound demonstrated stable mechanical mitral valve function. Following a multidisciplinary discussion, the decision was made to continue the pregnancy and maintain her warfarin regimen.

**Figure 2:**
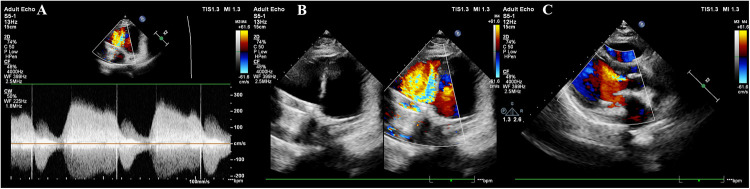
Cardiac ultrasound findings at 32 weeks of gestation. **(A)** Mechanical mitral valve with anterograde peak velocity (Vmax) of 2.4 m/s and mean pressure gradient (PGmean) of 6 mmHg; Tissue Doppler imaging shows mitral annular diastolic motion (e′ > a′). **(B)** Mechanical tricuspid valve with stable frame but restricted motion of one leaflet, demonstrating accelerated anterograde flow (Vmax = 2.7 m/s, PGmean = 17 mmHg). No significant perivalvular abnormalities are observed. The aortic valve appears thickened with hyperechoic echoes and incomplete closure. **(C)** Thickened aortic valve with hyperechoic echoes, incomplete closure, and mild regurgitation.

At 33 + 2 weeks, the patient's symptoms worsened, with dyspnea occurring after minimal activity. A repeat echocardiogram revealed suboptimal motion of one tricuspid valve leaflet, accelerated forward flow (Vmax = 2.7 m/s, PGmean = 17 mmHg), and possible subvalvular tissue proliferation, raising concerns for mechanical valve dysfunction(Figure 2). Given her Class III cardiac function and high-risk status (WHO Class IV-V), the multidisciplinary team recommended pregnancy termination via cesarean section. Warfarin was discontinued, and bridging therapy with LMWH and intramuscular vitamin K was initiated to achieve an INR <1.4. The patient and her family consented to cesarean delivery with intraoperative monitoring and with contingency plans for valve replacement if intraoperative dysfunction was confirmed.

At 33 + 3 weeks, she underwent a cesarean section under general anesthesia, delivering a live male infant (41 cm, 1880g, Apgar scores 5-8-8). The newborn was transferred to the pediatric ICU, with no cardiac abnormalities observed on ultrasound, and was discharged after 13 days. Intraoperatively, the patient's hemodynamics remained stable, and transesophageal echocardiography confirmed normal tricuspid valve motion. No immediate valve replacement was performed.

**Figure 3:**
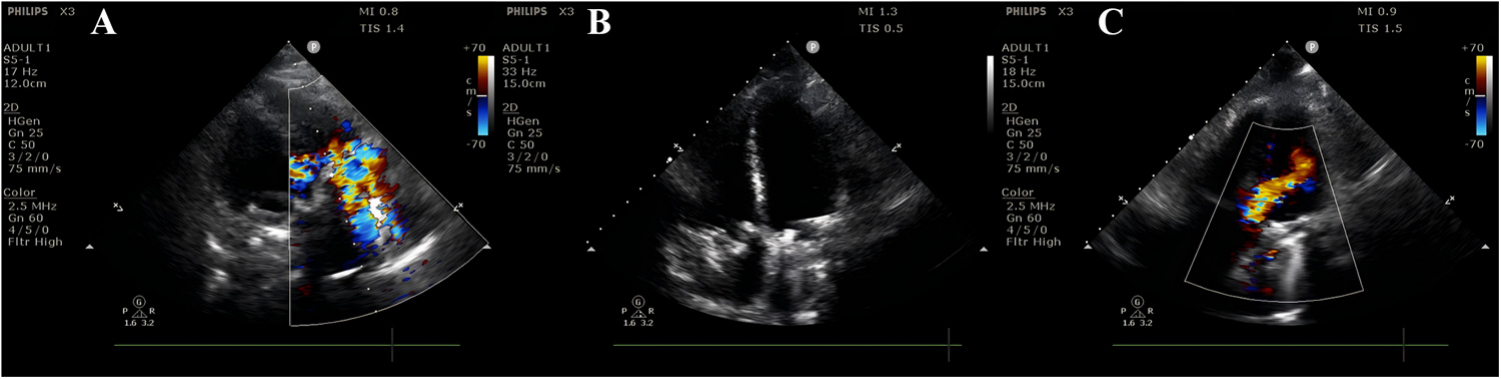
Postpartum cardiac ultrasound findings on day 3. **(A)** Mechanical tricuspid valve with significantly accelerated anterograde flow (Vmax = 2.5 m/s, PGmean = 14 mmHg) and severe regurgitation at the prosthetic orifice. **(B)** Mechanical tricuspid valve with stable frame; both leaflets exhibit semi-open positions (one leaflet shows no significant motion, while the other has restricted mobility). Hypoechoic echoes adherent to the valve surface (suspected thrombus or other etiology?). **(C)** Mechanical mitral valve with mildly accelerated anterograde flow. No definitive perivalvular regurgitation is observed. Color Doppler demonstrates clear flow signals without abnormal perivalvular echoes.

Three days after the cesarean, echocardiography revealed a semi-open mechanical tricuspid valve with restricted leaflet motion, weak echoes suggesting thrombus, and severe regurgitation, confirming mechanical valve dysfunction (Figure 3). After a multidisciplinary discussion, she underwent bioprosthetic tricuspid valve replacement on postoperative day 4 under extracorporeal circulation. Intraoperative findings included pericardial adhesions, right heart enlargement, thrombus on the mechanical valve, and restricted leaflet motion. The mechanical valve and subvalvular pannus were excised, and a bioprosthetic valve was implanted. The surgery was successful, and the patient recovered well.

## 3 Discussion

### 3.1 Maternal hemodynamic challenges

During pregnancy, the maternal circulatory system undergoes significant changes characterized by increased total blood volume, elevated cardiac output, higher heart rate, and reduced systemic vascular resistance. These hemodynamic alterations can exacerbate diastolic dysfunction in patients with valvular heart disease. The coexistence of pregnancy and cardiovascular disease represents a major contributor to maternal morbidity and mortality, ranking as the second leading cause of maternal death in China and the most common non-direct obstetric cause of death. Cardiovascular diseases complicate 1%−3% of pregnancies and account for 10%−15% of maternal deaths (12). In recent years, with the advancement of medical technology, an increasing number of reproductive-aged women with congenital or acquired heart disease are achieving pregnancy viability. This demographic shift presents substantial clinical management challenges (3).

In developing countries, rheumatic heart disease tends to occur at a younger age compared to developed regions. The European Registry of Pregnancy and Heart Disease reported that mitral stenosis and/or regurgitation is the most common site of valvular lesion (63%), followed by the aortic valve involvement (23%). Maternal mortality rates are substantially higher in pregnancies complicated by valvular heart disease vs. congenital cardiac conditions (13). A clinical study from Africa documented 50% mortality among women with severe symptomatic mitral stenosis during pregnancy, predominantly occurring in the postpartum period (14).

Patients with valvular heart disease must undergo a comprehensive evaluation before pregnancy. Those with severe valvular heart disease may undergo replacement surgery with a prosthetic mechanical valve. Prosthetic valve thrombosis is a rare complication in non-pregnant patients, with an incidence rate of approximately 0.1%–5.7% (15). In pregnancy, the risk of thrombosis increases due to the hypercoagulable state, raising the risk to about 10% (15). Compared to open-heart surgery, low-dose thrombolytic therapy guided by transesophageal echocardiography has a definite therapeutic effect on pregnant women with mechanical mitral valve thrombosis (16). It results in fewer maternal and infant complications, and there have been no reported cases of maternal mortality (17). The neonatal mortality rate is 20% (18). Once valve replacement is accepted, anticoagulation therapy is crucial. When formulating an anticoagulation treatment plan, it is necessary to consider comprehensively factors such as the type and location of the valve, history of thromboembolism, presence of atrial fibrillation, and the patient's compliance.

### 3.2 Fetal risks associated with anticoagulation therapy

Warfarin remains the most effective anticoagulant for mechanical heart valves in non-pregnant patients. However, during pregnancy, its transplacental passage poses significant fetal risks. In the first trimester, warfarin exposure has been associated with dose-dependent embryotoxic effects, including skeletal malformations (e.g., nasal hypoplasia, facial anomalies) and severe central nervous system defects (e.g., intraventricular hemorrhage, cerebral edema) (19, 20). In the late stages of pregnancy, warfarin use may result in neonatal intracranial hemorrhage, including subarachnoid hemorrhage and periventricular necrosis (21–23). A 2023 meta-analysis quantified this dose-response relationship: live birth rates were 83.6% for daily warfarin doses ≤5 mg vs. 43.9% for >5 mg, while congenital malformation rates increased from 2.3%–12.4% (24). Notably, these observational data face inherent limitations: warfarin is predominantly used in high-risk pregnancies (e.g., mechanical valve recipients), where underlying maternal pathology itself contributes to adverse outcomes. Interspecies differences in warfarin teratogenicity and ethical considerations regarding maternal-fetal safety have also limited the availability of high-quality evidence on its use during pregnancy, leaving warfarin's safety profile inadequately characterized despite clinical urgency. In contrast, LMWH demonstrates superior fetal safety, with a 92% live birth rate in pregnancies managed exclusively with LMWH (24). Nevertheless, its variable thromboprophylactic efficacy perpetuate clinical equipoise. This therapeutic dilemma underscores a critical gap: no anticoagulant optimally balances maternal and fetal safety throughout all gestational stages.

### 3.3 Pharmacologic strategies and evidence review

The 2014 American College of Cardiology and American Heart Association (ACC/AHA) guidelines (4) and the 2018 European Society of Cardiology (ESC) guidelines (21) recommend that patients with mechanical valve replacement continue low-dose warfarin anticoagulation (≤5 mg/day) during early pregnancy. When the daily dose exceeds 5 mg, transitioning to LMWH or unfractionated heparin (UFH) is advised, with dose adjustments guided by anti-Xa level monitoring. Notably, the ESC guidelines demonstrate a critical divergence: given to the increased risk of thrombosis during pregnancy, they do not recommend LMWH use at any gestational stage and consider warfarin to be the safest option, particularly when the daily dose remain below5 mg (25, 26). In summary, regardless of the anticoagulant chosen in early pregnancy, warfarin therapy is generally recommended during mid to late pregnancy. It is advised to discontinue warfarin and switch to LMWH or UFH prior to a planned vaginal delivery. In cases requiring urgent delivery during late pregnancy (e.g., acute valve dysfunction or hemodynamic instability), cesarean section is recommended when inadequate time exists for safe anticoagulant transition from warfarin to LMWH or UFH, thereby minimizing peripartum hemorrhagic risks associated with traumatic delivery (27). A meta-analysis published in 2023, which included 15 studies with 722 pregnancy events (87.2% mechanical valves, 12.5% biological valves), reported a maternal mortality rate of 1.33% and an overall bleeding risk of 6.9%. The risks of thromboembolism and miscarriage after mechanical valve replacement during pregnancy were 4.71% and 29.29%, respectively. Additionally, a warfarin dose >5 mg/day was associated with an increased incidence of adverse fetal events (28).

During pregnancy, the occurrence of mechanical valve dysfunction, regardless of the cause, poses a serious threat to both maternal and fetal safety. When maternal mechanical valve dysfunction is combined with heart failure, the pathophysiological mechanisms leading to impaired uterine-placental blood flow are multifaceted, directly threatening fetal oxygen supply and nutrient exchange. Firstly, mechanical valve dysfunction (such as thrombosis or valve leaflet dysfunction) leads to increased right ventricular afterload, reduced right heart output, and systemic venous congestion, significantly decreasing maternal cardiac output. During late pregnancy, maternal cardiac output needs to increase by 30%–50% compared to the non-pregnant state to maintain high placental perfusion (29). However, in right heart failure patients, this demand cannot be met, resulting in reduced uterine artery blood flow, which is reflected in increased pulsatility index (PI) and resistance index (RI) (30). Studies have shown that for every 0.1 increase in uterine artery PI, fetal birth weight decreases by approximately 200 g on average (31), which aligns with the phenomenon observed in this case, where the preterm infant (1880g) is below the average birth weight of a full-term infant despite being appropriate for gestational age.

The indirect effects of right heart failure further exacerbate placental underperfusion. Elevated central venous pressure impedes uterine venous return, increasing placental interstitial pressure and reducing maternal-fetal exchange efficiency. Meanwhile, sympathetic nervous activation and overactivation of the renin-angiotensin-aldosterone system (RAAS) counteract the vasodilatory effects of pregnancy hormones (such as estrogen), thereby aggravating uterine artery vasoconstriction. Furthermore, the additive effect of pulmonary arterial hypertension (PAH) limits blood volume redistribution, prioritizing perfusion to vital organs like the brain and kidneys. In contrast, placental perfusion is sacrificed, potentially leading to fetal hypoxic vascular remodeling (32). While this compensatory mechanism may maintain fetal survival in the short term, it may increase the risk of cardiovascular diseases during childhood in the long term (33, 34).

Coagulation abnormalities play a dual role in this process. On the one hand, anticoagulant imbalance (such as coagulopathy due to hepatic congestion) increases the risk of microthrombi in the placenta (35). On the other hand, the choice between warfarin or LMWH directly affects fetal safety (36). In this case, the use of low-dose warfarin (target INR 2.5–3.0) during early pregnancy may have minimized embryotoxicity, with reported teratogenic rates of 2.3% for low doses compared to 12.4% for higher doses. Transitioning to LMWH in late pregnancy helped reduce the risk of fetal intracranial hemorrhage. This case highlights the critical role of pharmacological interventions as key environmental factors influencing fetal outcomes.

### 3.4 Recommendations for multidisciplinary management

While prolonging gestational age can improve neonatal short- and long-term outcomes and quality of life, patient conditions must be thoroughly evaluated, and gestational age extension should be carefully timed under multidisciplinary management. The management of pregnancy complicated by maternal mechanical valve dysfunction demands a nuanced approach to safeguard fetal health, as exemplified by this case; central to fetal protection is the optimization of maternal hemodynamics and placental perfusion, emphasizing the need for vigilant monitoring of uterine and umbilical artery Doppler parameters to detect placental insufficiency early.

Anticoagulation strategy is pivotal in balancing maternal thrombotic risks and fetal safety. Transitioning from warfarin (INR 2.5–3.0) to LMWH at appropriate gestational weeks mitigated fetal intracranial hemorrhage risk while addressing maternal hypercoagulability. Currently, there is a lack of specialized guidelines and large-scale clinical studies to inform decision-making, necessitating comprehensive evaluations to integrate NYHA class and PAH severity based on the patient's medical history, physical examination, and relevant diagnostic tests.

For patients with poor cardiac function and high suspicion of mechanical valve dysfunction, an active strategy of concurrent cesarean section and cardiac surgery should be pursued to ensure maternal and fetal safety, provided the institution is equipped for cardiopulmonary bypass. Multidisciplinary collaboration remains the cornerstone of care. Intraoperative echocardiography and postpartum valve replacement exemplified coordinated cardiac-obstetric interventions and anesthesiology to reduce maternal and neonatal mortality and optimize perinatal outcomes.

## Data Availability

The original contributions presented in the study are included in the article/Supplementary Material, further inquiries can be directed to the corresponding author.
